# Supplementary material for: A fast and robust Bayesian nonparametric method for prediction of complex traits using summary statistics
Source: PLoS Genet. 2021 Jul 26;17(7):e1009697. doi: 10.1371/journal.pgen.1009697 (PMC8341714; doi:10.1371/journal.pgen.1009697)
Supplement: Attachment — Submitted filename: Reviewer.pdf [file pgen.1009697.s002.pdf]

Dear Dr. Speed,

Thank you very much for handling our manuscript. We appreciate the positive comments from the reviewers as well as their concerns about SDPR. In this revision, we have performed additional analyses to address those concerns. The main work that has been done in our revision is summarized below:

1. We have added LDpred2, Lassosum, and DBLSMM to the list of methods for comparison and showed that SDPR still performed well compared to these more recently developed methods.
2. We have derived an analytical formula to show that when two SNPs are genotyped on different individuals, the correlation of their effect sizes in the summary statistics would generally be less than what is expected in the reference panel. This somehow justifies the use of our modified likelihood function. We also performed a simulation to show the robustness of our method in this setting.
3. We showed that SDPR still worked well after incorporating SNPs of large effects and those within the MHC region.

Please find below our point to point responses to reviewers' concerns below. We hope you and the reviewers will find this revised manuscript satisfactory.

Best,

Geyu Zhou

Hongyu Zhao

Reviewer #1: Zhou and Zhao present SDPR, a fast and robust Bayesian nonparametric method for building polygenic scores based on summary statistics. Lately, many Bayesian methods have been developed to derive polygenic scores based on summary statistics as well. They all are very similar in the algorithm and implementation they use, with the exception that they assume different prior distributions. At first sight, SDPR does not seem very different.

Therefore there are a few comments/questions I would like the authors to address so that I can better understand what SDPR adds to the current literature. Then and only then could I recommend SDPR to be published in PLOS Genetics.

Major comments (in no particular order):

- I first want to congratulate the authors for sharing their analysis code on GitHub.

We appreciate the reviewer for this comment.

- The authors claim that their method is more robust to different architectures because they use Dirichlet processes. Before PRS-CS claimed the same thing by introducing continuous shrinkage priors and SBayesR by using a point-mass at 0 and a mixture of 3 Normal distributions (instead of using only one in e.g. LDpred). The authors present a hierarchical Bayesian framework similar (for non-Bayesian experts) to the one used in PRS-CS and present the Dirichlet process as an infinite Gaussian mixture model. Therefore I wonder how different SDPR is from PRS-CS and SBayesR. I would urge the authors to explain these differences in more detail and how they think this could make SDPR more robust to different architectures than other published methods.

We agree with the reviewer that Bayesian nonparametric is not easy to grasp, even from a Bayesian's perspective. Theoretically, Dirichlet process as an infinite Gaussian mixture model is able to approximate any continuous parametric distribution, thus including other published parametric distributions as special cases [1]. Similar to Figure 1 of DPR paper, we have added an illustrative example to show that the density of Dirichlet process prior adapts well to the density of normal distribution (LDpred-inf), point normal mixture distribution (LDpred/LDpred2), and three-point normal mixture distribution (SBayesR) (Supplementary Figure 2). Compared with SBayesR, our prior does not constrain the relationship between three non-zero normal variance components. Different with PRS-CS, our prior explicitly incorporates variable selection by setting the first variance component to 0, making it more flexible to shrink effects in a sparse setting. Simulation studies show that Dirichlet process indeed performs better than PRS-CS in the sparse setting and SBayesR in the polygenic setting. Therefore, we believe that Dirichlet process prior is more robust to different architectures.

- Sometimes I wonder if allowing too much flexibility in the model is really a good thing? I guess more flexibility can hinder convergence or even make fitting diverge as it is sometimes the case in SBayesR. Is this why LDpred works better when summary statistics come from a GWAS with a small sample size? I guess that SDPR would work only with GWAS summary statistics for large sample size then? The authors could comment on this to expand the Discussion.

We agree with the reviewer that allowing too much flexibility in the model sometimes hinders the convergence. We indeed observed such convergence issues, similar to some cases in SBayesR, when testing SDPR on real GWAS summary statistics in the early stage of method development. We investigated and found that the discrepancy between summary statistics and reference panel was the cause of the convergence issue and proposed a modified likelihood function to deal with it.

As for the second question, based on our experience, we believe that when the sample size is small, the noise and heterogeneity of GWAS summary statistics poses more challenge for methods trying to learn every parameter from data (PRS-CS auto, LDpred2-auto, SBayesR and SDPR). Under such circumstances, it is advantageous for methods like LDpred/LDpred2 to use an independent validation dataset to select the optimal parameters. We have expanded the discussion section to include the above points (second paragraph of the discussion section).

- Again, maybe a naive question, but the authors present their method as nonparametric, but I see that many parametric distributions are assumed as priors for everything. What does “nonparametric” mean here? How is SDPR less parametric than e.g. LDpred2-auto which just assumes that beta ~ N(0, h2/(Mp)), p~U(0,1) and estimate h2=beta^T R beta?

Nonparametric means our prior is adaptive to different parametric assumptions made by other published methods. Please refer to our response to comment 2 for more details. To answer the second question, LDpred2 assumes the same point normal mixture distribution as LDpred, and thus still makes a specific parametric assumption.

- The authors say they “refine the commonly used likelihood assumption to deal with the discrepancy between summary statistics and external reference panel”. In my opinion, it is a very interesting point of this paper. However, the authors use simulation scenarios that do not allow to show this at all. Clear comparisons that show how this change makes SDPR more robust to the discrepancy between summary statistics and external reference panels would be well received. An ultimate test (which might be too difficult) would be to use a reference panel from individuals of e.g. South Asian ancestry, or at least a mixture of European and South Asian ancestry.

We would like to thank the reviewer for this comment. We first analytically derive that when the individuals for genotyping of two SNPs are different, the correlation of the marginal effect sizes in the GWAS summary statistics will be less than the correlation in the reference panel, depending on how many individuals are overlapped ($N_{s,ij}$) for two SNPs ($cor = \frac{R_{ij} N_{s,ij}}{\sqrt{N_i N_j}} \leq \frac{R_{ij} \min(N_i, N_j)}{\sqrt{N_i N_j}} \leq R_{ij}$).

As an extreme example, for two SNPs that are in perfect LD ($R_{ij} = 1$), if they are genotyped on completely nonoverlapped individuals ($N_{s,ij} = 0$), the correlation of their effect sizes in the summary statistics would be zero. In reality where GWAS summary statistics are usually obtained through meta-analysis, we do not know $N_{s,ij}$ unless extra information regarding the sample size and inclusion of each study is available for each SNP. Thus, our modified likelihood function $\hat{\beta}|\beta \sim N(R\beta, \frac{R}{N} + aI)$ somehow protects against such discrepancy in a general way as it shrinks the correlation of effect sizes of two SNPs from R_{ij} to $\frac{R_{ij}}{1+aN}$.

We then performed a simulation to test whether SDPR, with modified likelihood function, is robust to this scenario. We used the same simulation setting as Scenario 1B (sample size 50,000), with 50% of SNPs genotyped on 80% of individuals. Similar to what we observed in the real data analysis, SBayesR encountered a convergence problem for MCMC, while SDPR with modified likelihood function converged and performed well (N = 50,000, Na = 0.25; R2 = 0.422).

Finally, we would like to note that even within a single ancestry and perfectly matched reference panel, the correlation of effect sizes in the summary statistics may not agree with the true LD status, which could be challenging for methods relying on accurately specifying such information. For the cross-population setting, we do not expect SDPR to work well since LD pattern can be different across different populations. Consequently, as already stated in the discussion section, we believe that future work to explicitly model the cross-population LD matrix would be more beneficial.

- On the same point, is quality control not enough to control the discrepancy between summary statistics and external reference panel? E.g. the ones proposed in LDpred2 (https://doi.org/10.1093/bioinformatics/btaa1029) or in DENTIST (https://doi.org/10.1101/2020.07.09.196535), or maybe even better, using both.

We would like to thank the reviewer for bringing the recent work of quality control to us. Indeed, how to deal with these issues is still an open research question. We are not able to run DENTIST after encountering an open issue (https://github.com/Yves-CHEN/DENTIST/issues/8) at the time of this revision, so we focus mainly on QC proposed in LDpred2. In our opinion, while the quality control proposed by LDpred2 does detect problems of sample size, it has potential drawbacks. First, as acknowledged by the author of LDpred2, the formula is not very accurate for meta-

analysis. Second, it does not directly address the issue of the discrepancy between summary statistics and external reference panel, as no LD information regarding the reference panel is used. For example, as discussed in the last comment, if two SNPs have the same correct sample size, but they are genotyped on different individuals, then they will not be removed by the QC criteria proposed by LDpred2 but will cause convergence problems for methods like SBayesR. Therefore, we believe that it is better to first use our modified likelihood function, then apply QC criteria proposed by LDpred2 or DENTIST if the convergence problem is not solved.

- Again talking about robustness of methods, the authors remove the MHC region, which is usually performed for methods that are not robust to long-range LD regions. Is it the case for SDPR? How much signal is lost when removing this region? SNPs with extremely large effects are also removed, which should be the ones that are most useful for prediction. I wonder then if SDPR is really a robust method. By the way, these two removals of SNPs should never be called “quality control”.

Our design of the new LD block partition algorithm ensures that long-range LD regions would not be divided into smaller blocks. There are many long-range LD regions in the human genome, and we did not remove them for our analysis. Thus, we believe that SPDR is robust to long-range LD regions.

We removed the MHC region following the quality control guidelines of LDhub, and for a fair comparison with SBayesR (which recommends removing MHC to avoid convergence issues). We agree with the reviewer that this may lead to the loss of signals. However, we believe it would not affect the performance on real traits except for IBD considered in our paper, since MHC region is more relevant to autoimmune and infectious diseases [2]. After adding SNPs within the MHC region back, we observed a slight increase of performance for all methods (~0.005 in AUC). SDPR still performs similarly with LDpred1 and other methods except for LDpred2 (Figure 2). We observed convergence issues for SBayesR after adding MHC, so we left its previous version unchanged.

We agree with the reviewer that SNPs with extremely large effects should not be removed as they are most useful for prediction. Initially, we did encounter convergence problems when running SDPR on lipid summary statistics due to those SNPs with extremely large effects. During this revision, we found that the cause of the convergence issue was due to the fact that most SNPs with extremely large effects were genotyped on a specialized metabolic chip in a separate cohort (~94K individuals). We then fitted the model using our modified likelihood function (5), and did not encounter convergence issues (Line 130-139 of main text). Compared with our previous results, the performance of all methods improved significantly after incorporating extremely large effects. SDPR still performed the best for HDL, LDL and TC, while PRS-CS performed the best for TG (Figure 2).

- A simulation scenario with p=0.1 (and even p=1) should be added since many traits are thought to be very polygenic.

We have added a simulation scenario of very polygenic setting (p = 1). As shown in the Figure 1 scenario 5, SDPR performed well in this scenario.

- Two methods that have been shown to perform very well in the literature, namely lassosum and LDpred2, are missing from the comparisons.

We have added Lassosum and LDpred2 to our comparison list. In the simulation, LDpred2 performed similar to SDPR and significantly better than LDpred, in particular when the sample size was large. Note that this is somehow expected since Scenario 1A, 1B, 1C and 5 satisfied the assumption of LDpred and LDpred-inf. SDPR performed equal or better than lassosum in all simulation settings. In the real data analysis, LDpred2 also performed significantly better than LDpred. Overall, SDPR still achieved the best performance for 6 traits (height, BMI, HDL, LDL, TC, BC), and top tier performance for 4 additional traits (IBD, T2D, SCZ, BP; within 0.003 of AUC difference compared with the top method). Furthermore, SDPR performed significantly better when compared with methods without the need of parameter tuning (PRS-CS auto, LDpred2 auto and SBayesR; Supplementary Table 9 and 10). Although SDPR did not perform best for TG and CAD, we still believe SDPR is a competitive method, especially given its easiness to use in terms of its computational advantage and no requirement for a validation dataset.

Minor comments:

- Figures could be made nicer and more readable by using ‘+ theme_bw(16)’ (or even 18 if necessary).

We thank the reviewer for this suggestion. We have adopted this advice to make the figure nicer.

- Maybe report the AUC of the PGS only, not of the full model, for better comparison of methods, and starting the y-axis at 0.5.

We thank the reviewer for this suggestion. We now used the barplot with the same scale on y axis, instead of boxplot, to display the performance of each method in the real data analysis. We also reported the AUC of PGS only for diseases. This indeed allows for a better comparison of methods.

- In the introduction, what does “reparameterization” refer to here?

Reparameterization (also known as parameter expansion or overparametrization) refers to writing $\beta = \eta\gamma$, assigning the normal prior on η and inverse gamma prior on γ, instead of directly assign a prior on β. This technique was introduced to accelerate the convergence of Gibbs sampler and avoid the influence of inverse gamma hyperparameters on the estimation of variance in a normal hierarchical model [3, 4].

- Is correction for genomic control really necessary if using Z-Scores instead of p-values?

When uncorrected z scores (or uncorrected beta and standard deviations) are available, it is not necessary to correct for genomic control. In fact, we used Z-scores, instead of p-values, in summary statistics of lipid traits because p values were adjusted for genomic control. However, sometimes even Z-scores had been corrected for genomic control, as we observed for the BMI summary statistics. In the latter case, it is necessary to account for genomic control, otherwise the effect sizes would be underestimated.

References:

1. Lijoi A, Prünster I, Walker SG. On Consistency of Nonparametric Normal Mixtures for Bayesian Density Estimation. Journal of the American Statistical Association. 2005;100(472):1292-6. doi: 10.1198/016214505000000358.
2. Matzaraki V, Kumar V, Wijmenga C, Zhernakova A. The MHC locus and genetic susceptibility to autoimmune and infectious diseases. Genome Biology. 2017;18(1):76. doi: 10.1186/s13059-017-1207-1.
3. Gelman A. Prior distributions for variance parameters in hierarchical models (comment on article by Browne and Draper). Bayesian Anal. 2006;1(3):515-34. doi: 10.1214/06-BA117A.
4. Gelman A, van Dyk DA, Huang Z, Boscardin JW. Using Redundant Parameterizations to Fit Hierarchical Models. Journal of Computational and Graphical Statistics. 2008;17(1):95-122. doi: 10.1198/106186008X287337.

Reviewer #2: The manuscript describes a summary statistics based non-parametric method, SDPR, for genetic prediction of complex traits. The authors applied SDPR through four simulation settings and applications to twelve traits in the UK Biobank. In the simulations, SDPR works quite similarly as SBayesR but outperforms the other methods. In the UK Biobank applications, SDPR outperforms the other methods in more than half of the examined traits. Overall, I think this is a very nice method that adapts DPR to summary statistics and large-scale data applications. It is a timely contribution to the PRS field. The developed method has the potential to be widely used. I only have a few main comments:

1. In our experience, the four compared methods (PRS-CS, SBayesR, LDpred, P+T) are not among the most accurate PRS methods and are usually quite easy to outperform. It would be useful to add comparisons with lassosum and DBSLMM, both of which work quite well across a range of settings.

We thank the reviewer for this suggestion. We have added lassosum and DBSLMM for comparisons. In simulation. Both lassosum and DBSLMM performed better than LDpred, but not as well as SDPR (Figure 1). It seems that DBSLMM is more suitable in the polygenic setting and lassosum is more suitable in the sparse setting. In the real data analysis, SDPR also outperformed lassosum and DBSLMM in most traits (Figures 2 and 3).

2. As a related note, I am a bit surprised to see that SDPR performs similarly as SBayesR in all simulation settings. It would be helpful to identify some simulation settings where SDPR can clearly outperform SBayesR. SDPR is a polygenic model that assumes all SNPs to have non-zero effects, while SBayesR is a sparse model that sets a proportion of SNPs to have zero effects. Therefore, it might be helpful explore a few polygenic settings where all or a large fraction of SNPs have non-zero effects. This way, the new simulation results will become well aligned with the current real data results.

We thank the reviewer for this suggestion. We have added a simulation scenario of very polygenic setting ($p = 1$). As shown in Figure 1 scenario 5, SDPR performed well in this scenario and better than SBayesR.

3. Given that SDPR is a summary statistics version of DPR, it would be beneficial to compare SDPR with DPR in some small-scale simulations. These simulations can help benchmark the computational gains brought by SDPR over DPR and evaluate the potential accuracy loss in SDPR as compared to DPR.

We have added a small-scale simulation setting to compare with SDPR using 10,000 individuals and 58,432 SNPs on chromosome 1. The effect sizes were generated under the mixture of Dirichlet delta and three normal distributions with total heritability fixed as 0.3. We fitted DPR model with four components and 5000 MCMC iterations, and SDPR model after generating summary statistics. The average R2 of DPR was 0.227, while the average R2 of SDPR was 0.204 (Supplementary Figure 3). DPR took about 3.5

hours and consumed 10.4 Gb of memory to finish MCMC, while SDPR took only 10 minutes and used 1.1 Gb of memory. This demonstrated the improved computational efficiency of SDPR over DPR without loss of much prediction accuracy.

Minor comment:

1. On line 56-357 on page 20, the authors mentioned that "However, these methods either require external training datasets or do no account for LD". This statement on the ref 9 (DPR) does not appear to be accurate. As far as I am aware of, DRP does not require an external training dataset and does account for LD; it just does not model summary statistics as SDPR does.

We thank the reviewer for this comment. We agree with the reviewer that DPR does not require an external training dataset. We have modified our original statement as "However, these methods either require access to individual-level data (DPR), external training datasets (NPS), or do no account for LD (So's method)" (line 510-511).

Reviewer #3: The authors present a non-parametric PRS based on a new idea for data-driven adaptive modeling of the underlying effect size distribution. Extending on the previous work on Dirichlet process regression, which required individual-level genotype data, they propose a new MCMC algorithm that allows for the training of a PRS with summary-level GWAS data and reference LD panel. The performance of the new approach is benchmarked in simulated and real data. The work is solid and will be of great interest to PLoS Genetics readership if the following concerns are addressed.

Simulation scenarios: I am somewhat disappointed that the new method does not outperform SBayesR in simulation. Does SDPR outperform SBayesR when the effect sizes are sampled from a distribution that is very different from the BayesR model? SDPR performs very robustly in real data; however, it's difficult to know whether this robustness comes from the flexible non-parametric prior or some other features of the algorithm.

We have added a simulation scenario of very polygenic setting (p = 1). As shown in Figure 1 scenario 5, SDPR performed well in this scenario and better than SBayesR. Therefore, we believe that SDPR canoutperform SBayesR when the effect sizes are sampled from a very polygenic distribution. We also added a simulation scenario to show that SDPR performed better when the sample size for each SNP differs, while SBayesR did not perform well due to the convergence issue. In the real data analysis, we believe that both the prior of SDPR and our design of the robust likelihood function would contribute to the robustness of the performance.

PRS-CS: I think PRS-CS authors share codes to prepare custom LD matrices upon request. Comparisons to PRS-CS are particularly important here because it is more similar to SDPR than SBayesR is. I agree that the Dirichlet process would be more adaptive and flexible than PRS-CS's Strawderman-Berger prior on \sigma^2, but it needs to be shown clearly in which conditions this leads to higher accuracy.

We followed the reviewer’s suggestion to use the code provided in this link (https://github.com/getian107/PRScs/issues/20) to prepare custom LD matrices based on UK biobank, making it the same as other methods and reran PRS-CS for all simulation scenarios. We found that the performance of PRS-CS slightly improved, but still a little underperformed compared with SBayesR and SDPR when the genetic architecture is sparse. We suspect there could be two reasons. First, PRS-CS uses the LD block provided by ldetect, which may inappropriately split the long-range LD regions into smaller blocks (Supplementary Figure 1A). This could lead to over-estimation if one SNP with large effect size falls in the long-range LD region. Second, PRS-CS puts an upper bound on \sigma^2 (\sigma^2 <= 1/N, Method part of PRS-CS paper). Although this improves the robustness and avoids the blowing up of effect sizes when there is discrepancy between summary statistics and reference panel, it may sometimes over-shrink SNPs with large effects. We also noticed that the performance of PRS-CS was better compared with other methods in the polygenic setting, as the above two points do not matter when there are a large number of SNPs with relatively small effect sizes in

each LD block. PRS-CS performed competitively in the real data analysis since most traits are likely to be polygenic.

Extremely large effects: the authors "excluded extremely large effects (z2 > 80) to improve the convergence of MCMC" (p. 10) along with MHC. I think this decision is understandable and practical but wonder if SDPR is less accurate in handling large effect tails compared to other methods.

We would like to thank the reviewer for this comment. We initially found including extremely large effects (z2 > 80) for lipid traits hindered the convergence of SDPR. During this revision, we found that the cause of the convergence issue was due to the fact that most SNPs with extremely large effects were genotyped on a specialized metabolic chip in a separate cohort (~94K individuals). We then fitted the model using our modified likelihood function (5), and no longer encountered the convergence issues (Line 130-139 of main text). Compared with our previous results, the performance of all methods improved significantly after incorporating extremely large effects. SDPR still performed best for HDL, LDL and TC, while PRS-CS performed best for TG (Figure 2). Therefore, we believe that SDPR is accurate in handling SNPs with large effect sizes.
