# Supplementary material for: A fast and robust Bayesian nonparametric method for prediction of complex traits using summary statistics
Source: PLoS Genet. 2021 Jul 26;17(7):e1009697. doi: 10.1371/journal.pgen.1009697 (PMC8341714; doi:10.1371/journal.pgen.1009697)
Supplement: Attachment — Submitted filename: response.letter.docx [file pgen.1009697.s003.docx]

Yale University

*Yale School of Public Health*

*School of Medicine*

*60 College Street*

*P.O. Box 208034*

*New Haven, Connecticut 06520-8034*

June 30, 2021

Dear Dr. Speed,

Thank you very much again for handling our manuscript. We appreciate you and the reviewers for your time and efforts to provide valuable comments which have improved the quality of our manuscript. In this revision, we have corrected some typos and addressed the technical concern raised by reviewer 2. We hope you and the reviewers find this revised manuscript satisfactory.

Sincerely yours,

Hongyu Zhao, Ph.D.

Ira V. Hiscock Professor of Biostatistics and Chair

Department of Biostatistics

Co-Director of Graduate Studies

Interdepartmental Program in Computational Biology and Bioinformatics

Yale University

Reviewer #1: I thank the authors for their revised work and do not have any further comment.
Just two typos:
- Hammond -> Hadamard
- ldtect -> ldetect

We thank the reviewer for the positive comment and careful reading. We have corrected these typos in the revised manuscript.

Reviewer #2: All my comments are well addressed and the paper is ready to publish. I just want to bring up one minor technical inconsistency between the github code and the main text. On line 177, the authors listed a set of hyper-parameter choices that were used for fitting DBSLMM, which implies the tuning version of DBSLMM was used. However, based on the github code, it seems that the automatic version of DBSLMM was used which estimates all hyper-parameters based on the training data automatically (since no validation data was used in the fitted code there). The automatic version has slightly worse performance than the tuning version, but comparing to either version is completely fine and would demonstrate the benefits of SDPR. I don't know if I went to the wrong github site; if not, it would be important to keep the main text consistent with the github code.

We thank the reviewer for the positive comment and careful reading our paper and code. The reviewer pointed out that the code on the github did not agree with the manuscript. We would like to clarify this point as we used a tuning version of DBSLMM with hyper-parameters listed in the manuscript. Based on the scripts of DBSLMM for simulation (<https://github.com/eldronzhou/SDPR_paper/blob/417507073d2f33fd23a2e3566a3a9753b75373cb/UKB_simulate/result/Scene1A/h2_0.5/50K/DBSLMM/dblsmm.sh#L23-L30>) and real data analysis (<https://github.com/eldronzhou/SDPR_paper/blob/417507073d2f33fd23a2e3566a3a9753b75373cb/UKB_real/BC/result/DBSLMM/dblsmm.sh#L17-L24>), it can be seen that hyperparameters pv and r2 were iterated as described. For simulations, we ran cv.R (<https://github.com/eldronzhou/SDPR_paper/blob/main/UKB_simulate/result/Scene1A/h2_0.5/10K/DBSLMM/cv.R>) to select the best parameter based on the validation dataset and calculated the score for the test dataset. For real data analysis, we first calculated the score for all individuals by the plink command (<https://github.com/eldronzhou/SDPR_paper/blob/417507073d2f33fd23a2e3566a3a9753b75373cb/UKB_real/BC/result/DBSLMM/dblsmm.sh#L29-L31>). We then performed the validation and testing procedure in a separate script for all methods (<https://github.com/eldronzhou/SDPR_paper/blob/417507073d2f33fd23a2e3566a3a9753b75373cb/UKB_real/BC/result/predict/getRes.R#L161-L175>). We hope these explanations clarify the issue and address the reviewer’s concern.
